# Trends in ExPEC serogroups in the UK and their significance

**DOI:** 10.1007/s10096-016-2707-8

**Published:** 2016-06-22

**Authors:** H. Ciesielczuk, C. Jenkins, M. Chattaway, M. Doumith, R. Hope, N. Woodford, D. W. Wareham

**Affiliations:** 1 Antimicrobial Research Group, Centre for Immunology and Infectious Disease, Blizard Institute, Bart’s and the London School of Medicine and Dentistry, Queen Mary, University of London, E1 2AT London, UK; 2 Antimicrobial Resistance and Healthcare Associated Infections (AMRHAI) Reference Unit, Public Health England, Colindale, UK; 3 Gastrointestinal Bacteria Reference Unit, Public Health England, Colindale, UK; 4 Centre for Infectious Disease Surveillance and Control, Healthcare Associated Infection and Antimicrobial Resistance Department, Public Health England, Colindale, UK

## Abstract

Extra-intestinal pathogenic *Escherichia coli* are a significant cause of urinary tract infection and bacteraemia within the UK. We sought to identify the serogroups of 658 *E. coli* isolates collected in the UK between January 2011 and March 2012, to better understand the ExPEC population and understand the relevance of serogroups in this pathotype. Isolates were typed and serogroup identified using established phenotypic and molecular methods. Sixty-two serogroups were identified; 54 among urinary isolates and 35 among bloodstream isolates. However, serogroups O25, O6, and O2 dominated both infection types. These serogroups were linked to the major ExPEC STs as follows: ST131-O25, ST73-O6, ST127-O6, and ST95-O2. The serogroup data from this study have increased our understanding of the ExPEC population in the UK, but also highlighted key ST–serogroup relationships within the major ExPEC clones. These data can be used to guide vaccine design and in the development of laboratory diagnostic tests targeting the ExPEC population.

## Introduction

Multi-locus sequence typing (ST) and phylogenetic grouping (phylogroup) have become established methods for typing extra-intestinal pathogenic *Escherichia coli* (ExPEC) strains. In the UK the ST131/B2, ST127/B2, ST95/B2, ST73/B2, and ST69/D lineages are the most frequently recovered from bloodstream infections (BSI) and urinary tract infections (UTI) [1–3]. However, prior to the widespread use of these molecular methods, serotyping was extensively used to characterise pathogenic *E. coli*, [4, 5] as well as to inform the development of *E. coli* vaccines [6].

In the UK, the incidence of *E. coli* BSI is increasing year on year (https://www.gov.uk), while *E. coli* remains the most common cause of UTI [7]. Therefore, a successful ExPEC vaccine has the potential to reduce the incidence of BSI and UTI attributable to dominant strains in the UK and around the world [8–10].

Over time a number of associations between serotypes, STs, and *E. coli* pathotypes have been observed, such as ExPEC O25b:H4 (ST131), enterohaemorrhagic *E. coli* (EHEC) O157:H7 (ST11), EHEC O104:H4 (ST678), and avian pathogenic *E. coli* (APEC) O1 (ST95) [11–14]. Serotyping remains an important method in identifying clinically-significant ExPEC strains and for the study of local ExPEC populations in laboratories lacking molecular capability [4]. However, few UK studies of ExPEC have evaluated the importance of serotype in relation to ST, phylogroup, and infection type [1–3, 15]. Therefore, we resolved to understand the relevance of serogroup within the UK ExPEC population and identify important links with infection type and sequence type.

## Materials and methods

### Bacterial strains

The 658 UK ExPEC isolates studied were part of a larger collection described previously [16]. They comprised 379 urinary isolates collected predominantly from patients in East London (Barts Health NHS Trust) and 279 bloodstream isolates collected as part of the BSAC Bacteraemia Resistance Surveillance Programme (2011). This included isolates from 38 centres located around the UK and Republic of Ireland [17]. The purpose of these two collections was to identify any common trends among ExPEC isolates recovered from urine versus isolates recovered from BSI.

The urinary isolates were recovered from episodes of complicated (*n* = 170, 45 %) and uncomplicated UTI (*n* = 161, 42 %), as well as cases of asymptomatic bacteruria (*n* = 48, 13 %). For bloodstream isolates, the presumed focus of BSI was genitourinary (*n* = 118, 42 %), gastrointestinal (*n* = 38, 14 %), chest (*n* = 14, 5 %), catheter-related line (*n* = 4, 1 %) or skin and soft-tissue infection (*n* = 2, <1 %), with the primary source unknown in 37 % (*n* = 103).

### Phenotypic serogrouping, phylogrouping and sequence typing PCR

Conventional serogrouping was performed using the method of Gross and Rowe [18]. Phylogroup was determined by PCR [19]. The major ExPEC STs (ST131, ST95, ST73 and ST69) were also identified by PCR [20], along with an additional primer pair to detect ST127. The forward (5′-CGCATAACAGGATTGTCTGG-3′) and reverse (5′-GCTATTCTACGGGCATTGTG-3′) primers for ST127 generated an amplicon of 404 bp.

## Results

### Regional distribution

The majority of study isolates (*n* = 445, 68 %) were collected in London (379 urinary and 66 bloodstream). Outside of London between four (South–East) and 34 (Yorkshire and Humber) bloodstream isolates were collected from each Public Health England (PHE) Surveillance region. In total, 203 (73 %) bloodstream isolates were collected from England, 21 (8 %) from Wales, 23 (8 %) from Scotland and 32 (11 %) from the Republic of Ireland. Patient demographics of the isolates studied are detailed in Table 1.

**Table 1:** Patient characteristics for all the study isolates

		Urinary isolates (%)	Bloodstream isolates (%)
Sex	Female	312 (82)	149 (53)
	Male	67 (18)	130 (47)
Age (years)	Range	1–99	0–97
	Median	39	73
Healthcare setting	Community-associated	337 (89)	190 (68)
	Hospital-associated	42 (11)	89 (32)
Total		379	279

The number of isolates is listed for each category, with the percentage in parenthesis

By region and country the same three serogroups dominated: O2, O6, and O25. It is noteworthy that O25 was identified most frequently, except in the North–East and East Midlands regions where serogroup O6 was the most common. In Wales this differed slightly, with O25 being the most common, followed by O16 and O75.

Phylogroup B2 was dominant in each country, accounting for 62–90 % of isolates, and was also the most prevalent phylogroup in each PHE region. It is noteworthy that B2 was the only phylogroup detected within the South–East and West Midlands, together with ST131 which was the only major ExPEC ST detected in these regions. ST131 also dominated in each country, except for Ireland where ST95 was marginally more prevalent. In addition to ST131, ST73 was the only other major ST detected in Welsh isolates. Similarly, Scottish isolates comprised mostly ST131, ST73, and also ST127. The dominant ST varied by PHE region between ST131 (East, Yorkshire & Humber, West Midlands, and the South), ST73 (North–East), ST95 (North–West and London) and ST127 (East Midlands).

### Relationship of serogroup to infection site

**Fig. 1:**
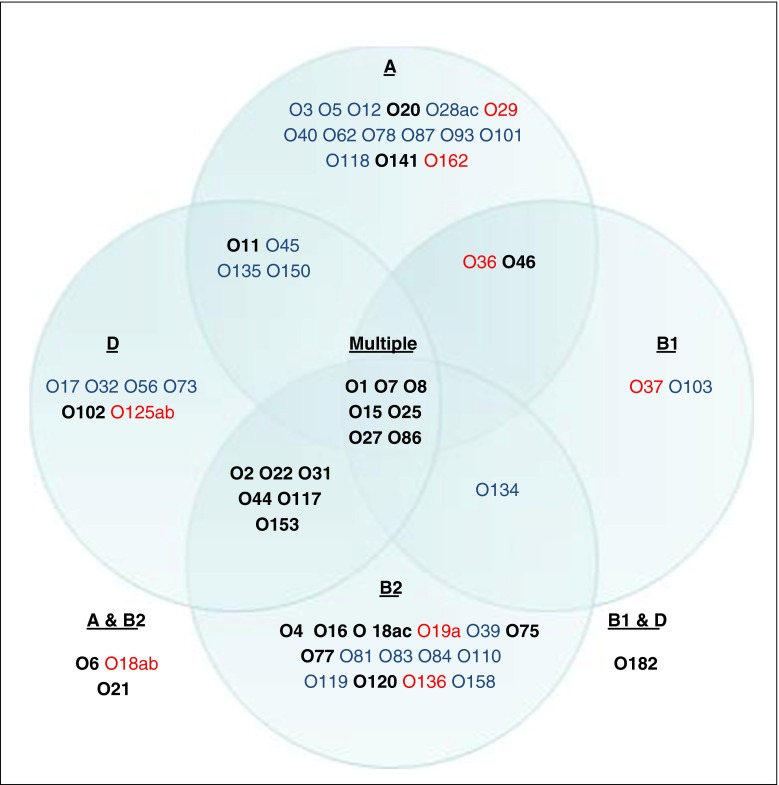
Serogroups identified in each of the four *E. coli* phylogroups. ‘Multiple’ lists the serogroups identified in three or four of the phylogroups. Only O86 was identified in all four phylogroups. Serogroups only identified in urinary isolates are coloured *blue*, serogroups only identified in bloodstream isolates are coloured *red* and serogroups identified in both are in *bold*. The most common serogroup in each of the phylogroups was O8 (A and B1), O25 (B2) and O44 (D)

There was greater serogroup diversity among the urinary isolates than the bloodstream isolates. Of the 62 serogroups identified, 54 were found in the urinary and 35 among the bloodstream isolates. Twenty-seven serogroups were specific to UTI, and eight were specific to bloodstream infections (Fig. 1).

The proportion of isolates comprising the most prevalent serogroups (O2, O6, and O25) differed by infection type. Twenty-nine percent (*n* = 110/379) of urinary isolates comprised these serogroups, with O6 the most commonly identified, followed by O25. This pattern was reversed in the 43 % of bloodstream isolates comprising these three serogroups (*n* = 120/279), with O25 the most commonly identified, followed by O6.

Antibiotic susceptibility data was performed as part of a previous study [16] and was reviewed for these three serogroups. Serogroup O25 was the most resistant of the three serogroups, followed by O2. Notably, BSI isolates within serogroups O6 and O25 were more resistant than the urinary isolates, but for O2 isolates this was reversed and the urinary isolates were more resistant than those from BSI.

### Relationship of serogroup to phylogroup

Phylogroups A, B1, B2, and D comprised 31, 10, 31, and 22 different serogroups respectively (Fig. 1). It is noteworthy that 19 serogroups were only detected in phylogroups A and B1, while 27 serogroups were only detected in phylogroups B2 and D (Fig. 1). Within all four phylogroups, the urinary isolates displayed a greater variety of serogroups than BSI isolates. However, in phylogroups B2 and D an even greater number of serogroups were identified in both infection types. The most common serogroups in each of the phylogroups were O8 (A and B1), O25 (B2), and O44 (D).

### Relationship between phylogroup and ST

The phylogroup and ST of the isolates studied are detailed in Table 2. ST131, ST95, and ST73 were only detected in phylogroups B2 and D, while ST69 was detected among all phylogroups except A, and ST127 was detected in phylogroups A and B2. With the exception of ST131, all other STs were identified more frequently in urinary isolates than BSI isolates.

**Table 2:** ExPEC phylogroup and sequence type for all study isolates

		Urinary isolates (%)	Bloodstream isolates (%)
Phylogroup	A	54 (14)	22 (8)
	B1	24 (6)	7 (3)
	B2	215 (57)	202 (72)
	D	86 (23)	47 (17)
	NK	0	1 (<1)
Major ST	ST131	41 (11)	74 (27)
	ST127	16 (4)	12 (4)
	ST95	56 (15)	34 (12)
	ST73	40 (11)	39 (14)
	ST69	50 (13)	14 (5)
	Other	176 (46)	106 (38)

One isolate was not assigned a phylogroup or a major ExPEC ST, but was identified as having serogroup O18ab.

### Relationship of serogroup to ST

The major ExPEC STs demonstrated reduced serogroup diversity, with just 29 of the 62 serogroups identified, as detailed in Table 3. ST131 and ST127 displayed least serogroup diversity (*n* = 5 each), while ST69 displayed the greatest diversity of serogroups (*n* = 15). With regard to infection type, both ST131 and ST73 had greater serogroup diversity within their BSI isolates than urinary isolates, whereas this pattern was reversed for ST95 and ST69, while ST127 isolates had equal distribution of serogroups within both infection types. Five serogroups were only identified in the major ExPEC STs: O17, O19a, O110, O125ab, and O136. Within the three most prevalent serogroups; ST95 comprised the majority of O2 isolates (*n* = 25/44, 57 %), and ST73 comprised more than half the O6 isolates (*n* = 42/75, 56 %), followed by ST127 (*n* = 21/75, 28 %). ST131 comprised the vast majority of O25 isolates (*n* = 89/104, 86 %).

**Table 3:** Serogroups identified in the major ExPEC sequence types by infection type

	ST131	ST127	ST95	ST73	ST69
Urinary		O16 O25	**O1** O6 O31 O110 O150 O153	O4 O158	O17 O45 O73 O102 O117 O150 O153
Bloodstream	O19a O136 O153	O11 O27		O8 O18ac O27	O27 O125ab
Both	**O16** **O25**	**O6**	**O2** O4 O16 O18ac	O2 **O6** O22 O25	O11 **O15 O25 O44** **O77** O86

Serogroups in bold text comprised >50 % of all isolates in that ST and the most common serogroups are underlined

## Discussion

This is the first large-scale UK study reporting on the serogroup diversity of *E. coli* causing UTI and BSI and the relationship between serogroup, phylogroup, and major ExPEC STs. Urinary isolates demonstrated greater serogroup diversity than bloodstream isolates, representing the ability of many *E. coli* strains to invade the bladder, but with fewer able to invade the bloodstream successfully [8]. Similarly, the less pathogenic phylogroups A and B1 comprised mostly urinary isolates, with 15 of the 19 phylogroup-specific serogroups (79 %) detected, while a smaller proportion of phylogroup-specific serogroups (*n* = 16/27, 59 %) were detected within the virulent phylogroups B2 and D bloodstream isolates.

In keeping with this pattern, the major ExPEC STs comprised mostly phylogroup B2 and D isolates, and displayed a limited repertoire of serogroups. This was notable for ST131/B2 (O25) and ST73/B2 (O6) which were prevalent among bloodstream isolates, while ST95/B2 (O2) and ST69/D (O77) were most frequently identified in urinary isolates. The link between phylogroup and major ExPEC ST is well established [21–23], but data from this study highlights that the relationship between serogroup and ST is just as important, as evidenced by the dominant serogroups found: ST131-O25 (B2), ST73-O6 (B2), and ST95-O2 (B2). This important link between serogroup and ST may have developed either through clonal expansion (ST131-O25) [13] or due to virulence-associated traits (ST127-O6 and ST73-O6) [24], but either way, identification of serogroup in *E. coli* isolates provides an alternative means to identify major ExPEC clones and understand the local ExPEC population.

While the serogroup repertoire for the major UK ExPEC STs may be limited, it appears to be changing when compared to previously published reports [1, 25]. This could represent the large sample size examined here or the fact that the UK ExPEC STs have evolved. Away from the major known STs, analysis of this UK collection identified 21 serogroups not previously documented among ExPEC isolates, again highlighting a limitation of studies that have focused on small populations or referred strain collections. These newly identified ExPEC serogroups included O12, O31, O32, O36, O37, O39, O40, O46, O56, O62, O81, O83, O87, O93, O110, O118, O135, O150, O158, O162, and O182 [4, 26, 27]. These novel ExPEC serogroups highlight the importance of fully understanding the local ExPEC population, clonal or otherwise, for future vaccine development, for genotypic laboratory tests targeting this particular microbial population, and to further understand the clinical importance and virulence of emerging strains of *E. coli* and the diseases they cause.

To conclude, this large study detailed the serogroup diversity of ExPEC isolates causing UTI and BSI in the UK, with particular reference to major STs. Data collected here can be used to expand our knowledge of ExPEC, as well as to inform vaccine development.
